# THE ART OF CARING: EXPLORING HOW DEMENTIA CARE PARTNERS MANAGE CARE CHALLENGES AND FIND RESILIENCE

**DOI:** 10.1093/geroni/igz038.2206

**Published:** 2019-11-08

**Authors:** Amanda N Leggett, Laura N Gitlin

**Affiliations:** 1 University of Michigan, Ann Arbor, Michigan, United States; 2 Drexel University, Philadephia, Pennsylvania, United States

## Abstract

Approximately 15 million Americans serve as family caregivers for a person with Alzheimer’s disease or another form of age-related dementia and this care can take a physical and emotional toll. Understudied is the process of how families actually provide care in response to care challenges, and how to find respite and resilience amidst care challenges. This symposium considers how caregivers handle daily challenges related to dementia including activities of daily living, behavioral and psychological symptoms of dementia, general health and medical comorbidities. In addition to characterizing care partner’s distinct styles of management (Leggett et al.) and knowledge and capacity to manage health care (Sadak et al.), the papers also provide perspective on positive aspects of care management such as the impact of respite on positive mood (Wylie et al.), the moderating role of relationship quality on responses to behavioral and psychological symptoms of dementia (Chunga et al.) and finally how caregivers’ problem-related, self-growth, and help-related behaviors compose their resilience to care challenges (Zhou et al.). To conclude, our discussant Dr. Laura Gitlin will offer insight on cross-cutting implications across studies and offer perspective on how research, intervention science, and clinical practice may better account for caregiver management styles to promote growth and resilience in caregivers and their care partners with dementia.

